# Direct age constraints on the magnetism of Jack Hills zircon

**DOI:** 10.1126/sciadv.add1511

**Published:** 2023-01-06

**Authors:** Richard J. M. Taylor, Steven M. Reddy, David W. Saxey, William D. A. Rickard, Fengzai Tang, Cauê S. Borlina, Roger R. Fu, Benjamin P. Weiss, Paul Bagot, Helen M. Williams, Richard J. Harrison

**Affiliations:** ^1^ Department of Earth Sciences, University of Cambridge, Downing Street, Cambridge CB2 3EQ, UK.; ^2^ School of Earth and Planetary Sciences, Curtin University, Bentley, WA 6102, Australia.; ^3^ Geoscience Atom Probe Facility, John de Laeter Centre, Curtin University, Bentley, WA 6102, Australia.; ^4^ Department of Earth, Atmospheric, and Planetary Sciences, Massachusetts Institute of Technology, Cambridge, MA 02139, USA.; ^5^ Department of Earth and Planetary Sciences, Harvard University, Cambridge, MA 02138, USA.; ^6^ Department of Materials, University of Oxford, Parks Road, Oxford OX1 3PH, UK.

## Abstract

A potential record of Earth’s magnetic field going back 4.2 billion years (Ga) ago is carried by magnetite inclusions in zircon grains from the Jack Hills. This magnetite may be secondary in nature, however, meaning that the magnetic record is much younger than the zircon crystallization age. Here, we use atom probe tomography to show that Pb-bearing nanoclusters in magnetite-bearing Jack Hills zircons formed during two discrete events at 3.4 and <2 Ga. The older population of clusters contains no detectable Fe, whereas roughly half of the younger population of clusters is Fe bearing. This result shows that the Fe required to form secondary magnetite entered the zircon sometime after 3.4 Ga and that remobilization of Pb and Fe during an annealing event occurred more than 1 Ga after deposition of the Jack Hills sediment at 3 Ga. The ability to date Fe mobility linked to secondary magnetite formation provides new possibilities to improve our knowledge of the Archean geodynamo.

## Teaser

Analysis of nanoscale clusters in Jack Hills zircon shows magnetization postdates deposition by more than one billion years.

## INTRODUCTION

Earth’s magnetic field undoubtedly played an important role in establishing the conditions necessary for the emergence of life on Earth (*1*), yet we know virtually nothing about the properties and behavior of the field during this time. The earliest paleomagnetic evidence for an active geodynamo comes from 3.5–billion year (Ga) rocks from the Barberton Greenstone Belt, South Africa (*2*, *3*). No undisputed paleomagnetic data exist before this time, leaving a gap of more than a billion years in the paleomagnetic record. Attempts to plug this gap have recently focused on the Jack Hills, Western Australia, the site of 2.65- to 3.05-Ga metaconglomerates containing detrital zircon grains that have been dated as far back as 4.4 Ga (*4*). Although zircon (ZrSiO_4_) is not intrinsically magnetic, these grains often contain magnetic inclusions that may have been trapped as the zircons grew within their parental granitic melt. The presence of magnetic inclusions makes zircon a potential target for single-crystal paleomagnetic analysis, subject to the following assumptions: (i) Each zircon crystal acquired a primary thermoremanent magnetization (TRM) during cooling of its parent granite; ii) a component of primary TRM survived pre- and postdepositional high-temperature metamorphism and low-temperature aqueous alteration; and iii) the component of primary TRM can be distinguished from all sources of secondary magnetization. If all these conditions are met, then the Jack Hills zircons have the potential to constrain the properties of the Hadean geodynamo. If any one of these conditions is violated, however, then the case for primary magnetization cannot be made.

Tarduno *et al.* (*5*) demonstrated the possibility of obtaining magnetic information from magnetite inclusions hosted within Jack Hills zircons that they dated between 4.4 and 3.3 Ga. However, this study was soon followed by intense debate over the interpretation of the results, including the nature of the inclusions themselves (*6*–*8*), the paleomagnetic data (*9*), and the use of conglomerate and microconglomerate tests to investigate thermal and chemical remagnetization of inclusions (*10*–*14*). The zircon crystal used in this study is one of the few Jack Hills grains confirmed to record a high-fidelity paleointensity record (*9*). Magnetic inclusions within this 3.979-Ga grain have, however, been interpreted as secondary features hosted within dislocations and nanoscale pores resulting from the accumulation and annealing of minor radiation damage (*7*). Since zircon contains very low levels of structurally bound Fe, the Fe required to form paleomagnetically significant amounts (*15*) of secondary magnetite must be sourced from the environment outside the grain itself and transported through micro- to nanoscale structures (*7*). The timing of the influx of Fe into the zircon therefore places important constraints on the growth of magnetite and the age of the resultant magnetization preserved within the zircon grain. However, previous studies have been unable to constrain the timing of these processes, and so, it has not been possible to discriminate whether magnetization took place shortly after zircon crystallization at ~4 Ga or much later.

**Fig. 1.**
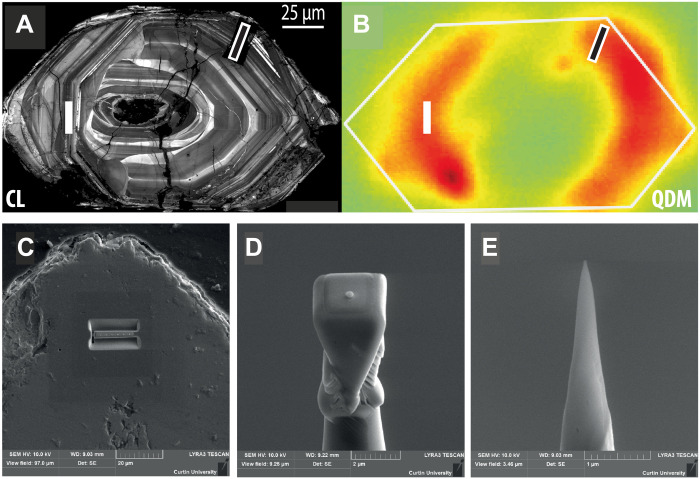
Location of the APT specimens relative to the microstructural and magnetic features of the zircon crystal. (**A**) Cathodoluminescence (CL) and (**B**) QDM images of the Jack Hills zircon grain. Magnetic portions of the grain are attributed to secondary magnetite formation. The target location for APT specimens is highlighted in (A) toward the left-hand section of the grain (white bar). The black bar in the top right of (A) shows the location of transmission electron microscopy samples studied in (*7*). (**C**) Location of the trench used to extract samples for APT. The six Pt dots mark the positions of each of the six extracted APT specimens. A single sample before polishing is shown in (**D**), and the final polished needle for APT is shown in (**E**).

Atom probe tomography (APT) provides a unique possibility to gain geochemical information on both trace element and isotopic abundances on small sample volumes and is most effective when used in conjunction with correlative techniques observing ever-decreasing length scales (*16*). Here, we present APT analyses of a previously studied zircon grain (*7*, *9*). The region targeted for APT analysis is a highly magnetic zone identified using quantum diamond microscopy (QDM)—a high-resolution magnetic imaging method that maps the remanent magnetic field on an intragrain length scale (Fig. 1). The source of this magnetic signal was identified to be secondary magnetite growing inside pores and along dislocation lines (*7*). Our APT data reveal trace element–enriched, nanoscale clusters within crystalline zircon from the highly magnetic zones. The elemental and Pb isotopic compositions of these clusters show distinct periods of cluster formation that can be used to place temporal constraints on the influx and remobilization of Fe and hence the formation of secondary magnetite.

## RESULTS

### Nanocluster characterization

**Fig. 2.**
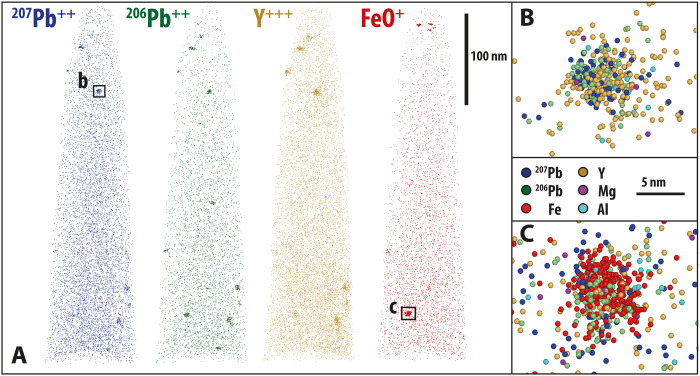
Representative examples of the APT data collected in this study. (**A**) Reconstructions of APT specimen data for a variety of key elements showing clustering of ^207^Pb^++^, ^206^Pb^++^, Y^+++^, and FeO^+^. Examples of Fe-absent and Fe-bearing clusters are shown in (**B**) and (**C**), respectively.

Visual inspection of the reconstructed atom probe data shows that cluster compositions are variable (Fig. 2, fig. S1, movies S1 to S6, and table S1). In previous studies of Jack Hills zircons, clusters were defined by the isoconcentration of Y because cluster compositions were consistent throughout each specimen (*17*). This approach is not valid for variable cluster compositions. Hence, to ensure that all clusters in atom probe specimens were selected for further analyses, clusters were defined and digitally extracted from the main dataset using the combined counts for Y, Yb, Pb, Mg, and FeO. Isolating and extracting the cluster data in this way has the advantage of significantly reducing the background signal within the clusters. A total of 94 clusters and a single dislocation region were identified.

All clusters were Pb bearing, enabling the ^207^Pb and ^206^Pb counts to be used to calculate a ^207^Pb/^206^Pb isotope ratio for each cluster. This isotopic composition reflects that of the zircon host at the time of cluster formation and can be used to calculate the age of cluster formation if the initial crystallization age of the zircon is known (*17*). The total number of Pb atoms in each cluster (broadly correlating with cluster volume) varied from 3 to 433. Because counting statistics mean that clusters with <30 Pb atoms yield large uncertainties in Pb isotope ratios, only a subset of 52 clusters with >30 counts of total Pb was investigated for statistical analysis of the geochemical signatures (table S1). Measured ^207^Pb/^206^Pb ratios from these 52 clusters range from 0.19 to 1.05. Comparison of these ratios with Y + Yb concentrations indicates that clusters with high Y + Yb typically have higher Pb concentrations and higher ^207^Pb/^206^Pb ratios (figs. S2 and S3).

**Fig. 3.**
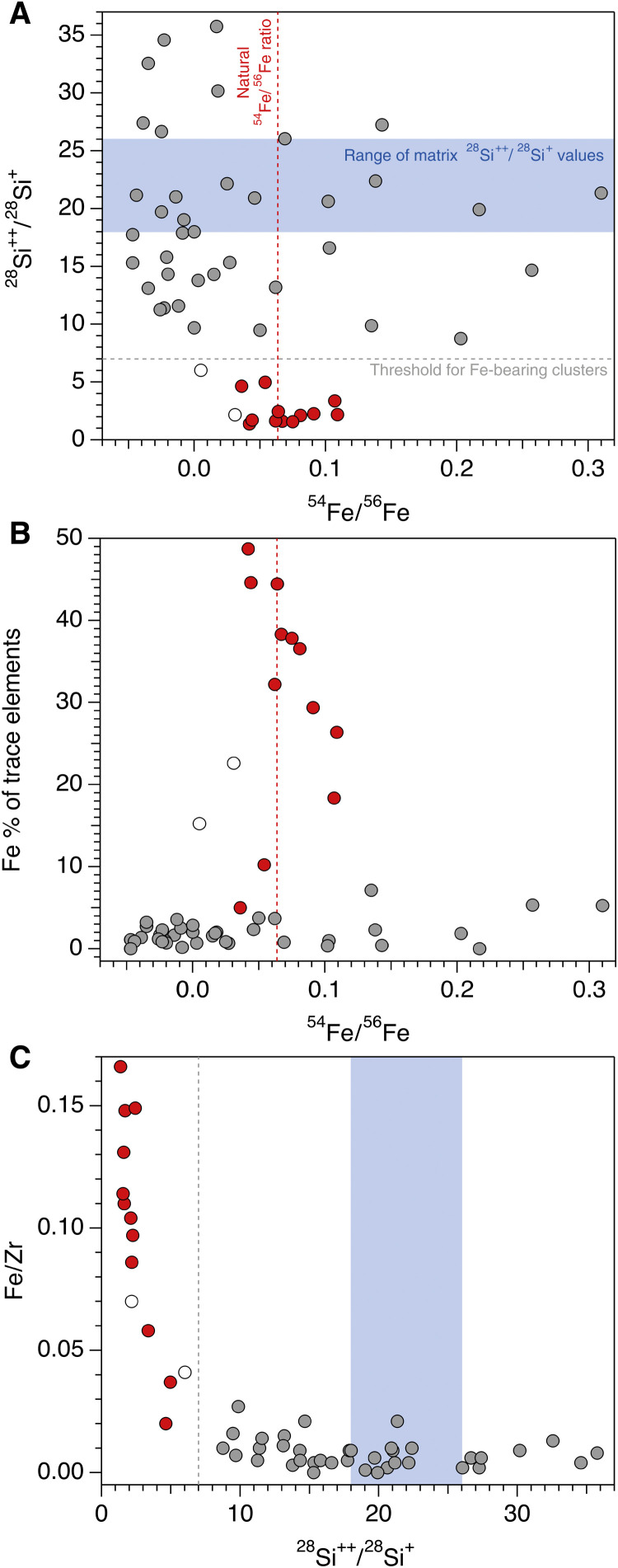
Classification of Fe-bearing clusters. (**A**) Plot of ^28^Si^++^/^28^Si^+^ and ^54^Fe/^56^Fe. These data provided the main discrimination for identifying a true Fe signal within the APT reconstructions. The ability to demonstrate that the APT Fe signal is truly above zero and not a ranging artifact is critical for interpreting the earliest timing of magnetite formation. Fe-rich clusters are shown in red. Blue range shows measured Si^++^/Si^+^ for the zircon matrix (fig. S4). Gray Fe-poor clusters show larger spread because of the small number of atoms within the clusters. (**B** and **C**) Major and trace element plots show a clear correlation between the values in (A) and discriminants such as Fe as a percentage of all trace elements (B) and Fe/Zr (C). However, these simpler parameters are unable to define the Fe-bearing clusters independently.

Although it is possible to identify the presence of Fe in some of the clusters based on Fe peaks in the APT data that do not have overlapping interferences (e.g., FeO^+^) (fig. S1), the quantification of Fe is complicated by mass/charge interferences related to the overlap between ^56^Fe^++^ and ^28^Si^+^ in the APT mass/charge spectra. To address this, a detailed investigation of multiple Fe isotopic peaks, alongside Si peaks at mass 28, is used to provide a discriminatory tool for determining the Fe concentration. The value of ^28^Si^++^/^28^Si^+^ varies systematically over the zircon APT analyses (fig. S4). This evolution of the charge state ratio most likely represents variation in the field over the duration of the analysis (*18*). However, the presence of doubly charged ^56^Fe^++^ increases the counts on the combined ^56^Fe^++^/^28^Si^+^ mass/charge peak, allowing a distinct reduction in the apparent ^28^Si^++^/^28^Si^+^ ratio to be used as a monitor for Fe (Fig. 3 and fig. S4). Furthermore, the doubly charged ^54^Fe^++^ peak at the nearby mass of 27 Da allows variations in the apparent ^54^Fe^++^/^56^Fe^++^ ratio to be used as a monitor of Fe, since the isotopic composition of natural Fe (comprising 5.845% ^54^Fe and 91.754% ^56^Fe) will yield a value of 0.064.

A plot of ^28^Si^++^/^28^Si^+^ versus ^54^Fe^++^/^56^Fe^++^ (Fig. 3A) demonstrates a threshold value for ^28^Si^++^/^28^Si^+^ of ~7, below which ^54^Fe/^56^Fe gives a well-constrained, robust mean value of 0.063 ± 0.015, and above which there is considerable scatter in Fe isotope ratios. This method separates the 52 clusters into 38 having no significant Fe component (pale gray filled circles) and 12 that are Fe bearing (red filled circles). Two clusters are intermediate in interpretation using these criteria (empty circles in plots). These distinctions are used to identify the clusters in all subsequent discussion of the data and figures. The identification of Fe-enriched clusters using both the Fe isotope ratio and Si peak interference (Fig. 3A) is further confirmed by increased Fe relative to total trace element enrichment in the clusters (Fig. 3B) and increasing Fe/Zr, where Zr is the major cation of zircon (Fig. 3C).

### Nanocluster formation

**Fig. 4.**
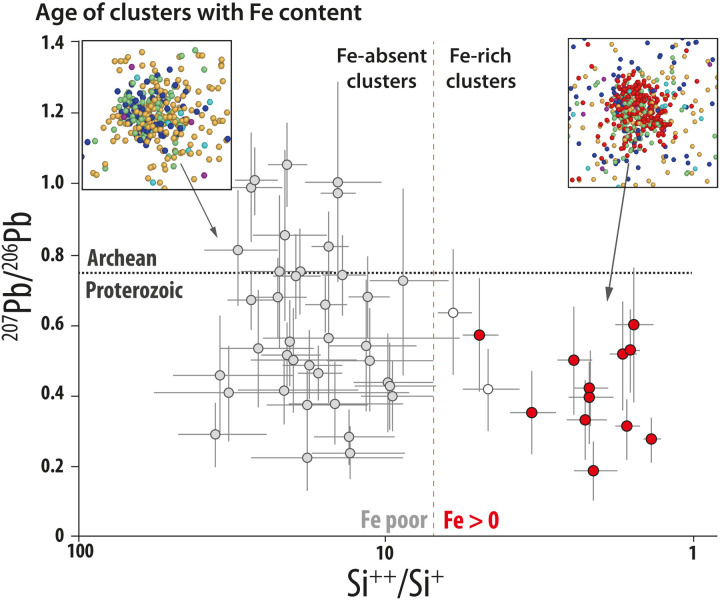
Age of clusters with Fe content. Plot of Pb-Pb ratio (representing the timing of cluster formation) versus ^28^Si^++^/^28^Si^+^ (a proxy for Fe content) for the 52 largest clusters analyzed across six APT specimens. The clear trend shows that the oldest Archean clusters are Fe-absent, and Fe-bearing clusters (red symbols) are only present in clusters with Proterozoic isotope ratios. The data suggest that Fe remobilization, required for magnetite formation, accompanied the second, younger clustering event at <2 Ga.

Analysis of the 12 Fe-bearing clusters reveals that they are exclusively low in Y + Yb (<100 counts) and also have low ^207^Pb/^206^Pb ratios (0.19 to 0.60) (Fig. 4 and figs. S2 and S3). Fe-absent clusters show almost the entire range of possible ^207^Pb/^206^Pb (0.23 to 1.05). However, some of these clusters have low Y + Yb (<100 counts) and have similar ^207^Pb/^206^Pb ratios to the Fe-bearing clusters. Hence, the cluster compositions define three end-member compositions, (i) high-^207^Pb/^206^Pb, high–Y + Yb, low-Fe clusters; (ii) low-^207^Pb/^206^Pb, low–Y + Yb, high-Fe clusters; and (iii) low-^207^Pb/^206^Pb, low–Y + Yb, low-Fe clusters. The similar ^207^Pb/^206^Pb ratios of the latter two cluster types (Fig. 4) are consistent with the simultaneous formation of these clusters with different trace element compositions. Support for this possibility comes from compositional heterogeneity and cluster-volume variations in single cluster-forming events in experimental studies of heat-treated zircon (*19*). Compositional heterogeneity is especially likely in this case due to the Fe being externally sourced (*7*).

## DISCUSSION

The clustering of ^207^Pb and ^206^Pb indicates the redistribution of radiogenic Pb. This has been interpreted to arise during high-temperature (~800°C) annealing of the radiation-damaged zircon lattice in natural samples (*17*, *20*) and has been recently confirmed experimentally (*19*). There is no work on the minimum temperatures of cluster formation, but it has been closely linked to the annealing of radiation damage, which may take place at temperatures of >250°C (*20*). Previous studies have also demonstrated the colocation of nanoclustered Pb with other compatible (e.g., Y and Yb) and incompatible (Al and Mg) solute ions (*4*, *17*, *19*, *21*), indicating that the formation of nanoclusters is an energetically favorable distribution of multiple trace elements within the zircon lattice.

The results indicate that the high-^207^Pb/^206^Pb, high–Y + Yb–bearing nanoclusters and low-^207^Pb/^206^Pb, low–Y + Yb clusters can be distinguished on the basis of their age of formation and their chemical composition, which is consistent with at least two discrete cluster-forming events. However, many of the clusters have compositions intermediate between the three identified end-member compositions. This could indicate the continuous temporal development of clusters between the high- and low-^207^Pb/^206^Pb end members. However, as discussed above, Pb cluster formation in zircon has been shown to form during discrete high-temperature annealing events and to be intimately linked to the recovery of accumulated radiation damage (*4*, *19*–*21*). Hence, cluster formation is likely to be kinetically limited during much of the zircon’s history, except for discrete periods of heating. Our preferred explanation is that some of the early-formed, high-^207^Pb/^206^Pb, high–Y + Yb, low-Fe clusters may have been compositionally modified by the addition of trace elements, including Pb, during the later cluster-forming event.

When Pb clusters form, they are separated from the U-bearing crystal lattice, trapping the distinct Pb isotope signature at the time of formation. The Pb isotope ratios of clusters measured by APT represent the time between the initial crystallization of the zircon grain and the separation of the cluster from the U- bearing crystal lattice (*4*, *20*). The ratios are not equivalent to the ^207^Pb/^206^Pb ratio measured by secondary ion mass spectrometry (SIMS) or thermal ionization mass spectrometry (TIMS) to determine the current age of the grain, since the analytical volume still contains a source of U. However, isotope ratios from Pb-bearing clusters in zircon can be used to constrain the timing of cryptic geological events (*4*, *20*).

**Fig. 5.**
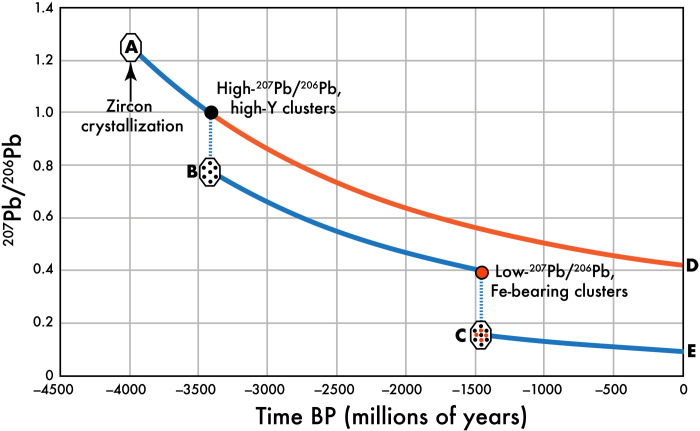
Pb evolution model indicating two end-member scenarios for Pb isotopic evolution of the studied zircon and associated Pb-bearing clusters. From the initial crystallization of the zircon at 3979 Ma ago, the isotopic composition of radiogenically produced Pb would evolve from point A along the blue line toward D. The ^207^Pb/^206^Pb ratio of ~1 in the high-Pb, high–(Y + Yb) clusters indicates cluster formation at ~3.4 Ga. In the case where Pb in the cluster represents a small fraction of the total Pb in the zircon, the matrix will continue to evolve along the orange line toward D. In this end-member case, the isotopic composition of the high-Fe clusters (^207^Pb/^206^Pb < 0.4) cannot be reached. In the case where all of the Pb was incorporated into the clusters, the matrix would have no Pb and the initial composition of radiogenically derived Pb would have an isotopic composition shown by point B. Extraction of Pb from this evolving zircon matrix to yield Pb clusters with ^207^Pb/^206^Pb < 0.4 would take place at ~1.5 Ga. Assuming that all of the matrix Pb migrated to the clusters at this time, the further evolution of the Pb isotopic composition of the zircon matrix would evolve from C to E. In situations falling between these two end-member cases, the age of cluster formation required to trap a ^207^Pb/^206^Pb of ~0.4 would be <1.5 Ga.

The analyzed zircon grain yields a concordant ion probe age, indicating that no significant Pb has been lost from the zircon grain since it crystallized 3979 million years (Ma) ago (*7*, *9*). The five clusters with the highest Y + Yb values yield ^207^Pb/^206^Pb ratios of 1.05 to 0.97. Given the crystallization age of the zircon, the mean ^207^Pb/^206^Pb ratio of these clusters would have evolved in the zircon by 3375 ± 160 Ma. This represents the time of extraction of the Pb from the U-bearing reservoir and is interpreted to date the formation of the older population of clusters (Fig. 5). This age not only is a major age peak in Jack Hills detrital zircon population (*22*) but also matches that of an Archean thermal event at ca. 3400 Ma previously determined from Y-bearing Pb clusters in Jack Hills zircon APT data (*4*). In this previous study, Pb-rich clusters were densely and homogeneously distributed in the atom probe specimen. Despite the similar age of the cluster-forming thermal event, Pb-rich clusters in the analyzed zircon grain are sparse and heterogeneously distributed. This difference most likely reflects differences in the amount of radiation damage in the different grains [cf. (*4*, *7*)] and shows that the nanoscale response of zircon to metamorphism may differ due to different microstructural states.

The ^207^Pb/^206^Pb ratios from the 12 Fe-bearing clusters overlap within the 2σ uncertainties of the counting statistics (table S1). Combining the lead counts from these 12 clusters yields a ^207^Pb/^206^Pb value of 0.397 ± 0.034 (1σ). For a zircon grain crystallizing at 3979 Ma, the decay constants of different U isotopes mean that this ratio cannot be achieved when U and Pb remain coupled. The Pb isotope composition of Fe-bearing clusters is therefore dependent on the Pb segregated to the 3.4-Ga clusters (Fig. 5). If all of the Pb that formed before 3.4 Ga migrates and is trapped in the older clusters, the U-bearing matrix will evolve over time to yield a ^207^Pb/^206^Pb ratio of 0.397 ± 0.034 at 1442 ± 123 Ma (1σ). If only a fraction of the Pb in the zircon was captured in the 3.4-Ga clusters, it would take longer for the zircon matrix to evolve to the Pb composition represented by the Fe-bearing clusters, and thus, the age of the cluster-forming event would need to be younger (Fig. 5). The age of 1442 Ma derived from the Pb isotopic composition of the Fe-bearing nanoclusters represents a maximum age for their formation. Therefore, even with the relatively large uncertainty associated with the low counting statistics of APT data, the Pb isotopic composition of the Fe-bearing clusters shows that Fe mobility must be <2000 Ma. This age coincides with several known thermal events that affected the northern margin of the Yilgarn craton (*10*) such as the 1960–2005 Ma Glenburgh orogeny, the 1780–1830 Ma Capricorn orogeny, and the emplacement of the Marnda Moorn and Warakurna large igneous provinces at ∼1210 and ∼1070 Ma, respectively.

Magnetite grains within the zircon have been demonstrated to be secondary features occupying distinct microstructural features within the crystalline zircon structure (*7*). The data reported here provide direct isotopic age constraints on the timing of Fe mobility associated with secondary magnetite formation and the recording of a TRM or thermochemical remanent magnetization (TCRM). Our data demonstrate that the externally sourced Fe, essential for the formation of magnetite, infiltrated the grain interior during the period between the first cluster-forming event at 3.4 Ga and the second event at <2 Ga.

The timing of Fe addition to the zircon grain is, however, difficult to precisely quantify. Although there is little evidence to support the addition of Fe to zircon during metamorphic events [e.g., (*20*)], previous studies have documented the addition of Fe to zircon during low-temperature alteration [e.g., (*23*, *24*)], and this seems to be enhanced by radiation damage (*25*). The first clustering event is likely to have healed any existing radiation damage, and so, a significant amount of time would be needed before any Fe would have been able to enter the crystal through radiation-enhanced alteration (*7*). However, the analyzed zircon grain is a detrital grain that went through the weathering and sedimentation cycle at some stage between the two thermal events recorded in the zircon by Pb-rich clusters. It seems most likely that low-temperature alteration associated with this sedimentary cycle was responsible for the addition of Fe to the zircon grain. The second clustering event led to the remobilization of Pb and Fe. We suggest that the formation of magnetite and the acquisition of TRM or TCRM primarily occurred during this younger clustering event, which would have led to the recrystallization of radiation-damaged zircon and the formation of magnetite according to the mechanism(s) proposed in (*7*).

We cannot entirely rule out the possibility that some formation of magnetite occurred as Fe infiltrated the zircon after 3.4 Ga, meaning that a component of chemical remanent magnetization could have been acquired during the period from 3.4 to <2 Ga. Either way, the magnetization recorded is at least 600 Ma younger than the crystallization age of the zircon and most likely associated with the <2-Ga event. Given that the Fe is most likely sourced from the sedimentary rocks from which the detrital grain was extracted, subsequent to the deposition of the sediment, the major component of magnetization reflects a field to which the Jack Hills sediment was exposed and not the precursor igneous rocks. Although this result may rule out the use of ancient zircons to constrain the intensity of the Hadean magnetic field, the ability of these crystals to acquire and retain high-quality paleomagnetic remanence data (*9*), combined with the ability of APT to track the influx of Fe and constrain the timing of magnetite formation, means that there is now a route available to start using zircon single-crystal paleomagnetism to fill major gaps in our understanding of the Earth’s geodynamo.

## MATERIALS AND METHODS

Atom probe specimens were prepared from a magnetic portion of the grain, identified as crystalline in backscattered electron imaging and within fine-scale oscillatory zoning in cathodoluminescence imaging (*7*) indicative of primary magmatic crystallization (*26*). A total of six atom probe specimens were prepared and analyzed using standard focused ion beam techniques (Fig. 1) (*27*). APT analyses were undertaken using a CAMECA local electrode atom probe (LEAP) 4000X HR at the Geoscience Atom Probe Facility, Curtin University. Full operating conditions are listed in table S2 following the recommendations in (*28*). Each specimen was run at a base temperature of 69 K under ultrahigh vacuum (10^−11^ torr) conditions. A high voltage was applied to the needle-shaped specimen, and field evaporation of atoms from the specimen tip was accomplished by focusing a pulsed (200-kHz) ultraviolet (λ = 355 nm) laser on the specimen apex. Laser power was set at 300 pJ. As the specimen radius increases during evaporation, the voltage applied to the specimen was increased from ~3 to 10 kV to maintain a constant ion detection rate of one ion every 100 pulses. Ions were sequentially recorded on a position-sensitive detector, and the composition was determined using time-of-flight mass spectrometry. APT measurements of individual atom probe specimens yielded between 51 and 77 million atoms during analysis.

Assignment of the mass/charge peaks obtained from APT analyses to elemental and molecular species was undertaken using standard ranging protocols for zircon (*19*, *29*). The three-dimensional (3D) distribution of the ranged peaks was then reconstructed for all six analyzed APT specimens (Fig. 2, fig. S1, and movies S1 to S6). All six specimens show the same main characteristics in the 3D reconstructions of the APT data. As expected for crystalline zircon, there is a uniform distribution of uranium (represented as UO in fig. S1). However, some trace elements (Al, Mg, Fe, Y, Yb, and Pb) show distinct segregation to 10- to 20-nm clusters with between 1 and 20 clusters per specimen.

Clusters were identified using a maximum separation method for cluster finding within APT data (*30*, *31*). Trace element mass peaks were selected for Y^+++^, Y^++^, Yb^++^, Pb^++^, Mg^++^, and FeO^+^, with clusters defined within the spatial reconstruction as combinations of these atoms having a maximum separation (*d*_max_) of 1.2 nm to their nearest neighbor (order *k* = 1), with at least 30 atoms in total (*N*_min_ = 30). Local major and other trace elements are then incorporated into each cluster using a “link” distance (*L*) of 0.6 nm and an “erosion” length (*d*_e_) of 0.3 nm.
