# Supplementary material for: Direct age constraints on the magnetism of Jack Hills zircon
Source: Sci Adv. 2023 Jan 6;9(1):eadd1511. doi: 10.1126/sciadv.add1511 (PMC9821853; doi:10.1126/sciadv.add1511)
Supplement: Supplementary file 1 — Tables S1 and S2 Figs. S1 to S4 [file sciadv.add1511_sm.pdf]

Science Advances
AAAS

Supplementary Materials for

Direct age constraints on the magnetism of Jack Hills zircon

Richard J. M. Taylor *et al.*

Corresponding author: Richard J. Harrison, rjh40@esc.cam.ac.uk

Sci. Adv. **9**, eadd1511 (2023)
DOI: 10.1126/sciadv.add1511

The PDF file includes:

Tables S1 and S2
Figs. S1 to S4
Legends for movies S1 to S6

Other Supplementary Material for this manuscript includes the following:

Movies S1 to S6

Supplemental Materials

Sample ID	Fe enriched	Zirconium counts			REE counts									Other trace element counts				Pb counts			Total REE	Total trace	Background (2 Da range near 28 Da)			$^{56}Fe^{++}$ and $^{28}Si^{+}$ peak overlap at 28 Da			$^{54}Fe^{++}/^{56}Fe^{++}$		$^{28}Si^{++}/^{28}Si^{+}$		$^{207}Pb/^{206}Pb$		Fe% of traces	Fe/Zr
		Zr	ZrO	ZrO_2	Y	YO	Dy	Ho	Er	Tm	Yb	Lu	LuO	Mg	Al	ScO	FeO	^{206}Pb	^{207}Pb	Total Pb	counts	counts	counts	counts/peak	1σ	$^{54}Fe^{++}$	$^{28}Si^{++}$	28 Da peak	ratio	1σ	ratio	1σ	ratio	1σ	%	ratio
M3	Y	161	1283	42	47	6	12	0	16	3	33	2	6	19	4	0	246	87	24	111	125	505	4	0.27	0.135	17	545	399	0.042	0.011	1.37	0.09	0.28	0.064	48.71	0.166
M2	Y	106	906	28	39	14	9	1	17	2	36	2	0	8	4	0	136	57	30	87	120	355	8	0.54	0.191	19	440	275	0.067	0.016	1.60	0.12	0.53	0.119	38.31	0.131
M2	Y	101	659	22	45	6	1	1	9	3	29	2	1	11	1	0	86	55	17	72	97	267	6	0.405	0.165	12	306	187	0.062	0.019	1.64	0.15	0.31	0.086	32.21	0.110
M3	Y	50	411	9	15	2	3	0	3	0	8	0	0	8	0	3	33	50	21	71	31	146	3	0.2025	0.117	3	195	90	0.031	0.020	2.17	0.28	0.42	0.109	22.60	0.070
M1	Y	39	201	9	3	2	3	0	4	1	4	0	0	16	1	0	5	43	18	61	17	100	0	0	0.000	1	130	28	0.036	0.036	4.64	0.97	0.42	0.118	5.00	0.020
M2	Y	85	637	30	48	4	6	0	15	0	99	6	2	8	0	3	28	35	20	55	180	274	7	0.4725	0.179	4	326	66	0.054	0.031	4.97	0.67	0.57	0.160	10.22	0.037
M5	Y	84	804	11	25	3	10	1	11	1	84	3	1	5	5	3	37	33	21	54	139	243	9	0.6075	0.203	1	442	74	0.005	0.014	6.01	0.76	0.64	0.178	15.23	0.041
M2	Y	56	570	18	21	0	5	1	7	0	27	0	0	5	0	2	95	33	17	50	61	213	6	0.405	0.165	7	256	151	0.044	0.018	1.70	0.17	0.52	0.154	44.60	0.148
M1	Y	49	394	12	5	0	1	0	10	0	11	0	1	9	0	0	68	32	16	48	28	153	8	0.54	0.191	6	209	86	0.064	0.030	2.44	0.31	0.50	0.153	44.44	0.149
M1	Y	43	286	10	8	0	7	1	3	0	5	1	0	8	0	2	29	33	13	46	25	110	4	0.27	0.135	6	115	53	0.109	0.049	2.18	0.36	0.39	0.129	26.36	0.086
M4	Y	40	288	14	10	0	0	0	5	0	16	1	0	8	2	1	20	34	12	46	32	109	3	0.2025	0.117	5	151	45	0.107	0.052	3.37	0.57	0.35	0.119	18.35	0.058
M1	Y	40	276	14	7	3	3	0	5	1	7	0	2	3	1	1	32	33	11	44	28	109	1	0.0675	0.068	6	146	65	0.091	0.039	2.25	0.34	0.33	0.116	29.36	0.097
M2	Y	33	278	15	6	0	1	0	2	0	9	0	0	3	0	0	34	32	6	38	18	93	4	0.27	0.135	5	124	59	0.081	0.040	2.11	0.33	0.19	0.083	36.56	0.104
M2	Y	44	336	15	6	1	0	3	4	1	16	0	0	9	1	1	45	20	12	32	31	119	0	0	0.000	6	125	80	0.075	0.032	1.56	0.22	0.60	0.219	37.82	0.114
M2	N	310	2482	110	249	57	30	6	72	16	466	28	23	9	0	2	18	216	217	433	947	1409	32	2.16	0.382	1	1251	49	-0.025	0.023	26.66	4.06	1.00	0.097	1.28	0.006
M2	N	317	1514	65	246	21	24	5	40	6	265	41	8	13	6	41	17	173	168	341	656	1074	30	2.025	0.370	3	946	68	0.015	0.027	14.31	1.85	0.97	0.105	1.58	0.009
M2	N	294	1887	96	265	47	31	7	68	15	358	35	13	8	2	14	20	156	164	320	839	1203	26	1.755	0.344	1	1100	54	-0.014	0.020	21.02	3.03	1.05	0.118	1.66	0.009
M1	N	222	1675	70	191	52	27	6	47	9	357	35	17	2	4	8	7	157	129	286	741	1048	23	1.5525	0.324	3	836	56	0.027	0.033	15.33	2.17	0.82	0.098	0.67	0.004
M4	N	205	1637	75	188	43	20	4	44	15	335	24	9	7	5	3	4	158	106	264	682	965	17	1.1475	0.278	5	733	28	0.143	0.089	27.25	5.47	0.67	0.084	0.41	0.002
M4	N	188	1502	59	104	35	20	3	40	15	355	21	19	4	7	4	9	158	104	262	612	898	14	0.945	0.253	0	713	46	-0.021	0.006	15.80	2.45	0.66	0.083	1.00	0.005
M3	N	174	1204	52	182	24	15	4	37	6	243	14	5	8	6	6	5	100	74	174	530	729	13	0.8775	0.243	1	568	42	0.003	0.025	13.79	2.25	0.74	0.113	0.69	0.003
M2	N	166	1358	58	165	42	18	7	30	13	241	12	14	4	1	4	10	82	81	163	542	724	14	0.945	0.253	0	660	25	-0.039	0.013	27.40	5.80	0.99	0.155	1.38	0.006
M2	N	208	1594	71	121	22	21	6	33	8	300	22	7	5	2	4	12	91	67	158	540	721	13	0.8775	0.243	0	693	36	-0.025	0.008	19.71	3.45	0.74	0.119	1.66	0.006
M3	N	169	1042	38	171	31	6	2	26	4	193	19	5	1	2	2	1	88	66	154	457	617	3	0.2025	0.117	0	472	25	-0.008	0.005	19.03	3.94	0.75	0.122	0.16	0.001
M4	N	202	1448	60	120	25	23	2	33	8	300	22	11	1	2	1	7	103	48	151	544	706	16	1.08	0.270	5	630	39	0.103	0.062	16.59	2.81	0.47	0.081	0.99	0.004
M3	N	179	1135	43	161	25	19	8	20	10	165	9	7	18	2	5	14	87	59	146	424	609	17	1.1475	0.278	0	558	50	-0.023	0.007	11.40	1.72	0.68	0.114	2.30	0.010
M3	N	184	1346	49	123	27	20	3	25	6	294	21	13	0	2	2	16	86	58	144	532	696	15	1.0125	0.261	5	650	30	0.138	0.082	22.39	4.33	0.67	0.115	2.30	0.010
M3	N	154	1042	46	117	10	19	4	27	8	147	9	4	7	2	3	12	93	48	141	345	510	14	0.945	0.253	2	483	24	0.046	0.063	20.91	4.55	0.52	0.092	2.35	0.010
M3	N	113	962	31	85	24	13	6	17	2	160	13	8	2	2	2	5	74	36	110	328	449	16	1.08	0.270	0	408	24	-0.047	0.016	17.75	3.90	0.49	0.099	1.11	0.005
M2	N	130	835	34	88	24	9	2	21	4	154	13	4	3	1	9	9	58	47	105	319	446	11	0.7425	0.224	1	431	15	0.018	0.072	30.18	8.34	0.81	0.159	2.02	0.009
M2	N	88	734	23	139	16	8	4	22	7	205	32	9	3	1	3	2	58	32	90	442	541	6	0.405	0.165	2	322	16	0.102	0.095	20.62	5.42	0.55	0.121	0.37	0.002
M2	N	35	251	2	15	0	1	0	0	0	5	0	0	2	0	0	6	60	25	85	21	114	3	0.2025	0.117	2	124	6	0.310	0.278	21.35	9.23	0.42	0.099	5.26	0.021
M4	N	37	269	11	13	1	1	0	7	0	17	0	0	8	4	0	5	55	22	77	39	133	3	0.2025	0.117	1	150	16	0.050	0.065	9.48	2.52	0.40	0.101	3.76	0.016
M4	N	32	286	10	21	3	1	0	5	1	10	1	0	8	3	0	5	60	17	77	42	135	3	0.2025	0.117	1	169	13	0.062	0.081	13.19	3.85	0.28	0.078	3.70	0.015
M5	N	28	312	12	15	0	0	0	4	0	14	1	0	1	3	1	3	56	21	77	34	119	1	0.0675	0.068	0	142	8	-0.009	0.009	17.89	6.56	0.38	0.096	2.52	0.009
M1	N	27	246	12	14	1	3	0	8	0	8	1	0	3	1	1	3	54	13	67	35	110	5	0.3375	0.151	0	127	10	-0.035	0.019	13.11	4.45	0.24	0.074	2.73	0.011
M3	N	38	221	10	12	1	3	0	5	1	8	0	1	5	0	1	2	42	18	60	31	99	0	0	0.000	0	126	13	0.000	-	9.69	2.82	0.43	0.121	2.02	0.007
M2	N	106	771	27	91	23	6	4	13	7	145	10	2	1	1	0	7	45	13	58	301	368	12	0.81	0.234	1	401	12	0.017	0.092	35.76	11.24	0.29	0.091	1.90	0.008
M4	N	24	185	8	8	2	2	0	0	0	5	2	0	8	2	0	1	36	18	54	19	84	3	0.2025	0.117	0	88	8	-0.026	0.018	11.26	4.26	0.50	0.144	1.19	0.005
M4	N	26	207	6	10	3	1	0	2	0	10	0	1	10	1	0	5	37	14	51	27	94	4	0.27	0.135	2	99	7	0.257	0.234	14.67	5.96	0.38	0.119	5.32	0.021
M1	N	36	191	7	26	4	0	2	4	1	15	1	0	2	9	0	0	34	17	51	53	115	5	0.3375	0.151	2	153	8	0.217	0.202	19.92	7.54	0.50	0.149	0.00	0.000
M3	N	56	361	13	113	14	3	2	16	3	70	4	1	1	2	2	2	25	25	50	226	283	4	0.27	0.135	0	197	14	-0.020	0.011	14.33	4.04	1.00	0.283	0.71	0.005
M2	N	101	666	31	88	13	6	1	12	4	145	4	3	0	0	3	3	27	23	50	276	332	10	0.675	0.213	0	325	16	-0.044	0.018	21.16	5.66	0.85	0.242	0.90	0.004
M3	N	59	395	13	44	10	3	0	18	3	95	6	3	2	0	0	2	28	21	49	182	235	11	0.7425	0.224	1	228	11	0.025	0.100	22.16	7.33	0.75	0.217	0.85	0.004
M1	N	30	293	14	15	2	4	0	6	0	11	0	0	27	6	0	9	32	14	46	38	126	2	0.135	0.095	2	137	14	0.135	0.108	9.87	2.80	0.44	0.140	7.14	0.027
M3	N	83	721	30	63	5	2	0	10	1	109	9	3	3	0	0	2	30	16	46	202	253	5	0.3375	0.151	1	252	10	0.069	0.107	26.05	8.69	0.53	0.165	0.79	0.002
M4	N	22	128	3	4	0	2	2	3	0	4	0	0	0	0	0	2	32	13	45	15	62	1	0.0675	0.068	0	63	2	-0.035	0.043	32.57	24.21	0.41	0.134	3.23	0.013
M4	N	10	132	5	6	1	3	0	2	0	0	0	0	3	2	1	0	25	14	39	12	57	2	0.135	0.095	0	44	3	-0.047	0.044	15.31	9.55	0.56	0.187	0.00	0.000
M3	N	25	184	6	13	0	2	0	4	0	6	1	0	1	3	0	2	31	7	38	26	70	0	0	0.000	0	72	4	0.000	-	18.00	9.25	0.23	0.094	2.86	0.009
M1	N	22	188	4	6	0	1	0	5	0	10	0	0	21	1	0	3	24	13	37	22	84	2	0.135	0.095	0	126	11	-0.012	0.010	11.58	3.69	0.54	0.187	3.57	0.014
M2	N	58	478	13	57	9	2	2	12	2	100	5	5	1	0	1	2	24	11	35	194	233	2	0.135	0.095	0	203	6	-0.023	0.019	34.59	14.66	0.46	0.167	0.86	0.004
M4	N	27	175	2	29	2	2	0	6	2	25	5	1	2	0	1	2	18	13	31	72	108	3	0.2025	0.117	3	121	14	0.203	0.137	8.76	2.51	0.72	0.263	1.85	0.010

Table S1

Summary of the chemical and isotopic composition of the 52 identified clusters having total Pb counts > 30. The ratio of ^{207}Pb and ^{206}Pb counts for each cluster are used without any background correction, since the background noise in the Pb peak ranges is very low within each cluster. The uncertainties in each isotope count are due only to counting statistics (±√N). The relative uncertainties in the isotope counts are then combined in quadrature to obtain the relative uncertainty in the isotope ratio. Background corrections are used in calculating the apparent $^{54}Fe^{++}/^{56}Fe^{++}$ and $^{28}Si^{++}/^{28}Si^{+}$ ratios, which include the potential peak overlap at 28 Da. Uncertainties are then calculated from counting statistics and propagated as for the Pb isotope ratios.

Specimen						
Sample ID	M1	M2	M3	M4	M5	M6
**Acquisition parameters**						
Instrument Model	LEAP 4000X HR					
Laser Pulse Energy (pJ)	300	300	300	300	300	300
Pulse Frequency (kHz)	200	200	200	200	200	200
Set Point Temperature (K)	60	60	60	60	60	60
Target Ion Detection Rate	0.01	0.01	0.01	0.01	0.01	0.01
Evaporation Control	Detection Rate					
Laser Wavelength (nm)	355					
Nominal Flight Path (mm)	382					
Chamber Pressure (Torr)	2.6E-11	2.4E-11	2.6E-11	2.5E-11	2.5E-11	2.6E-11
**Data Summary**						
Acquisition (LAS) Root version	15.41.342l					
Analysis Software	IVAS 3.8.2					
Total Ions	59,917,939	77,205,065	51,011,765	55,276,902	51,087,094	53,584,854
Single (%)	71.4%	71.6%	70.6%	71.1%	70.1%	70.8%
Multiple (%)	28.2%	28.0%	29.1%	28.6%	28.7%	28.9%
Partial (%)	0.4%	0.4%	0.3%	0.4%	0.4%	0.3%
Vol//bowl corr. Peak (Da)	16					
M/ΔM for $16O^+$	984	1018	1008	1021	1003	1005
$M/\Delta M_{10}$ for $16O^+$	425	448	437	439	423	433
Time-independent background (ppm/ns)	23.462	24.368	21.982	23.985	23.672	21.416
**Reconstruction**						
Reconstruction CAMECAROOT Version	18.46.428					
V initial; V final (kV)	4.9; 9.6	3.6; 10.0	3.7; 9.5	3.5; 10.0	3.5; 9.2	3.7; 10.0
Assumed E-field (V/nm)	32	32	32	32	32	32
Final specimen state	Intact	Intact	Intact	Intact	Intact	Intact
Pre-/post-analysis imaging	SEM/NA					
Radius evolution	Shank					
Field factor (k)	3.3					
Image compression factor	1.65					
Detector efficiency	36%					
Avg. atomic volume (nm^3/atom)	0.01076					

Table S2

Table S2. APT acquisition and reconstruction parameters for specimens M1 – M6 following recommendations of (*28*).

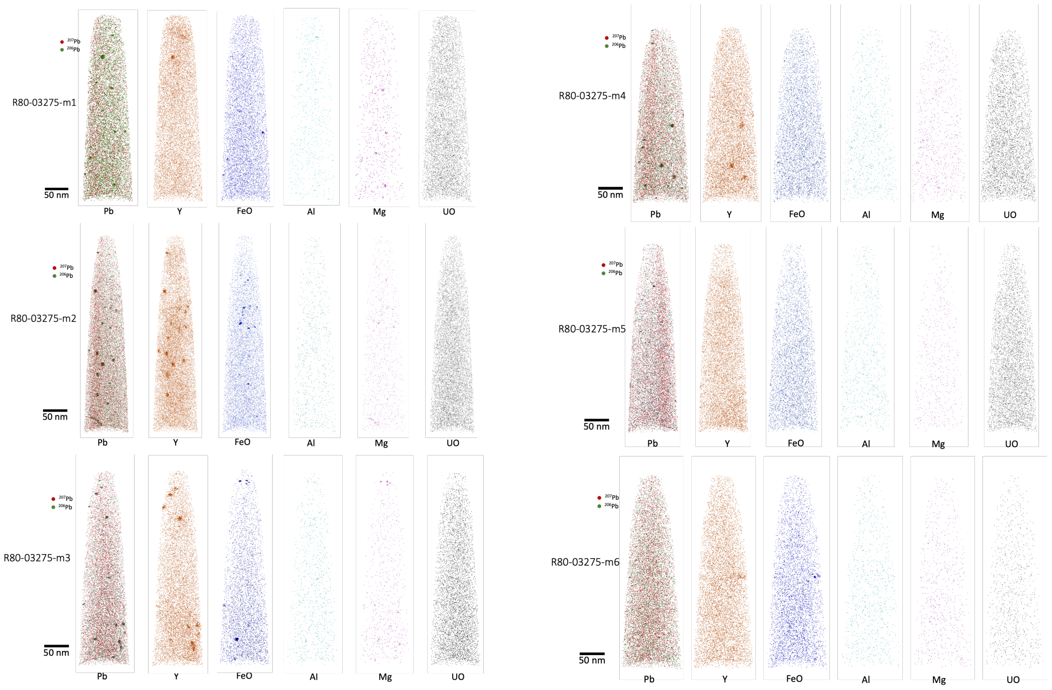

Figure S1

Figure S1. APT reconstructions of specimens M1 – M6 showing the ranged distributions of Pb, Y, FeO, Al, Mg and UO.

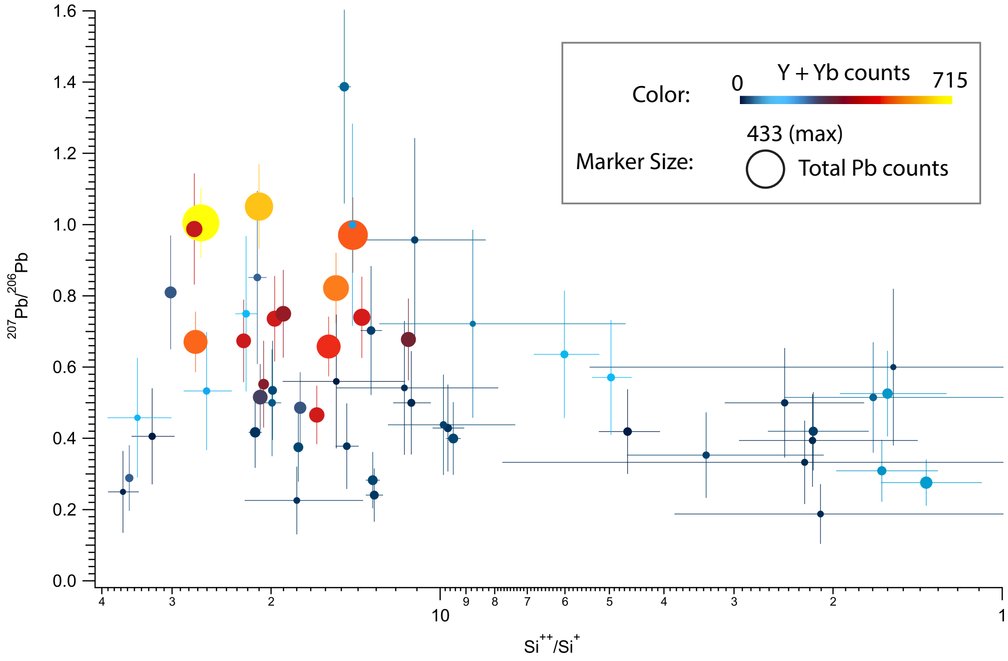

Figure S2

Figure S2. Figure showing variation in $^{207}Pb/^{206}Pb$ and Si^{++}/Si^{+} charge state ratio for clusters within the 6 Jack Hills zircon atom probe specimens (M1-M6). Color variations correspond to variations in (Y+Yb) counts and the marker size corresponds to total Pb counts.

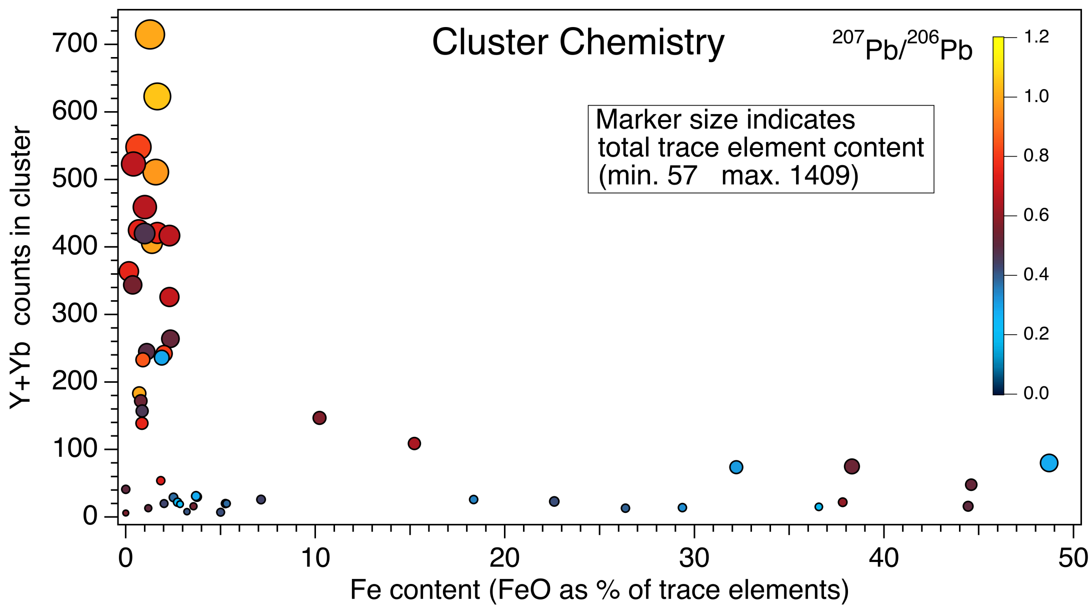

Figure S3

Figure S3. Figure showing variation in Y+Yb counts in each cluster versus FeO as a percentage of trace elements within the 6 Jack Hills zircon atom probe specimens (M1-M6). Color variations correspond to variations in ($^{207}Pb/^{206}Pb$) ratio and the marker size corresponds to the total trace element counts.

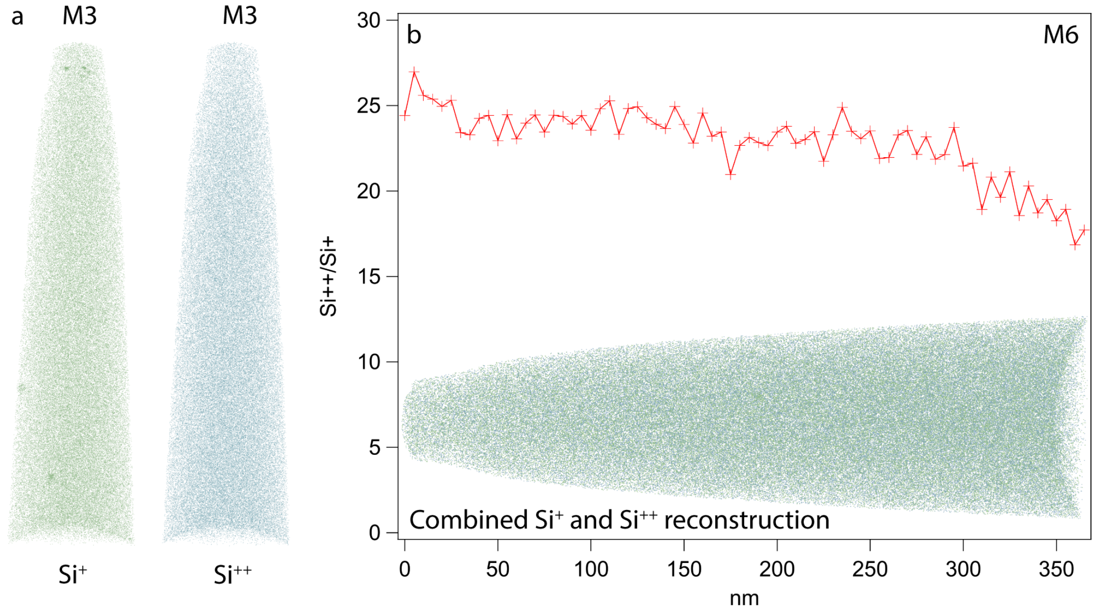

Figure S4

Figure S4. Si^{+} and Si^{++} data from two specimens (M3 and M6). a) Reconstructed APT data for Si^{+} (at 28 Da) and Si^{++} (at 14 Da). Clusters in the Si^{+} reconstruction coincide with FeO clusters in the same specimen (Fig. 2) allowing low Si^{++}/Si^{+} (<7) (Fig. 3a) to be used to identify Fe clusters. b) Evolution of Si^{++}/Si^{+} in specimen M6 as a function of distance through the specimen. Si^{++}/Si^{+} values are calculated from slices of 1M atoms taken along the specimen axis. In this case, distance represents the evolution of evaporation over time and the systematic change in Si^{++}/Si^{+} likely reflects changes in field evaporation conditions during the analysis. The range of Si^{++}/Si^{+} ~ 26-18 is significantly higher than that observed in Fe-rich clusters (<7).

Supplemental Movie 1. 3D animations of ^{207}Pb and ^{206}Pb reconstructions in atom probe specimen M1.

Supplemental Movie 2. 3D animations of ^{207}Pb and ^{206}Pb reconstructions in atom probe specimen M2.

Supplemental Movie 3. 3D animations of ^{207}Pb and ^{206}Pb reconstructions in atom probe specimen M3.

Supplemental Movie 4. 3D animations of ^{207}Pb and ^{206}Pb reconstructions in atom probe specimen M4.

Supplemental Movie 5. 3D animations of ^{207}Pb and ^{206}Pb reconstructions in atom probe specimen M5.

Supplemental Movie 6. 3D animations of ^{207}Pb and ^{206}Pb reconstructions in atom probe specimen M6.
